# Genome Sequence and Biochemical Properties of Bifidobacterium longum Strain ICIS-505, Isolated from the Intestine of a Healthy Woman

**DOI:** 10.1128/MRA.00491-19

**Published:** 2019-08-15

**Authors:** Sergey V. Andryuschenko, Elena V. Ivanova, Natalia B. Perunova, Oleg V. Bukharin

**Affiliations:** a Institute for Cellular and Intracellular Symbiosis of the Ural Branch of the Russian Academy of Sciences, Orenburg Federal Research Center, Orenburg, Russia; University of Rochester School of Medicine and Dentistry

## Abstract

This report describes the genome sequence of Bifidobacterium longum strain ICIS-505, isolated from human feces. The size of the genome was 2,448,844 bp (59.71% G+C content), including 3,751 bp of the crypto-plasmid pBL505. Annotation revealed 2,241 gene sequences, including 2,033 proteins, 7 rRNA genes, 76 tRNA genes, and 4 noncoding RNA genes.

## ANNOUNCEMENT

Bifidobacterium longum is a common member of the human gut microbiota and is frequently present at high numbers in the human gut microbiota throughout life, which is indicative of a close symbiotic host-microbe relationship (1, 2). The B. longum taxon currently recognizes four subspecies, *longum*, *infantis*, *suis*, and *suillum* (3–5). They are believed to exert various health-promoting effects; they produce various nutrients for their host, prevent infections caused by intestinal pathogens (6–8), and modulate a normal immunological response (9). Therefore, B. longum cultures are promising for their use in probiotics (10), although the precise mechanism of such activities has barely been studied. This knowledge gap has prompted research efforts, in particular, those focused on the comparative and functional genomics of *Bifidobacterium* spp. (11). Functional genome analysis of bifidobacteria is important for understanding how this species adapts to a particular niche. For example, more than 8% of the annotated genes found in the genomes of B. longum and Bifidobacterium breve are predicted to encode proteins involved in the complex plant-derived carbohydrate metabolism (12).

Here, we present a draft genome sequence of B. longum strain ICIS-505, isolated from the feces of a healthy 41-year-old woman from Orenburg, Russia.

B. longum strain ICIS-505 was initially isolated on a Schaedler agar plate (HiMedia Laboratories Pvt. Limited) from a 1.5-hour feces sample, which was diluted serially in 0.9 NaCl solution to 10^9^-fold by mass.

Strain ICIS-505 was initially identified as B. longum using the biochemical species identification kit ANAEROtest 24 (Lachema, Czech Republic). The taxonomic identity of this strain was verified on a cell protein profile using a matrix-assisted laser desorption ionization–time of flight (MALDI-TOF) mass spectrometry Biotyper system (Bruker Daltonik, Bremen, Germany) and using 16S rRNA gene sequencing.

For the DNA isolation procedure, a single colony of ICIS-505 agar culture was inoculated and cultivated in 4 ml of sterile Schaedler medium for 48 hours in a 0.6% oxygen and 5% carbon dioxide atmosphere at 37°C in a CO_2_-incubator (Binder, Tuttlingen, Germany). After incubation, the culture was centrifuged at 4,000 × *g* for 6 min. The pelleted cells were resuspended in 50 μl of Tris-buffered saline with 2 μg of hen egg white lysozyme (HEWL) and incubated at 37°C for 60 min. The suspension was mechanically homogenized using silica beads. DNAses were inactivated using proteinase K. The extracted DNA solution was purified with a standard phenol-chloroform extraction method (13) and precipitated using ethanol (14). The DNA sediment was dissolved in Milli-Q deionized water.

The genomic DNA of B. longum ICIS-505 was used to prepare a DNA library with the Nextera XT DNA sample preparation kit (Illumina, San Diego, CA). The library was sequenced in a 2 × 300-nucleotide run using the MiSeq reagent kit version 3 and the MiSeq desktop sequencer (Illumina). A total of 1,431,950 sequence reads were generated. The reads were quality trimmed using the sliding window mode of the Trimmomatic program using default parameters (15). *De novo* genome assembly was performed using the SPAdes genome assembler (St. Petersburg Genome Assembler) version 3.10.1 with default parameters (16). The assembly yielded 60 contigs covering a total of 2,448,844 bp with an *N*_50_ value of 224,635, a G+C content of 59.71%, and an average coverage of 56.8×. The genome sequence was annotated using the National Center for Biotechnology Information (NCBI) Prokaryotic Genome Annotation Pipeline (PGAP) (https://www.ncbi.nlm.nih.gov/genome/annotation_prok), which revealed 2,241 gene sequences, including 2,033 proteins, 121 pseudogenes, 7 rRNA genes (5S, 16S, and 23S), 76 tRNA genes, and 4 noncoding RNA (ncRNA) genes.

The revealed properties of B. longum ICIS-505 (e.g., a high level of acetate production) may be useful for probiotic development. This strain can serve as one of the models for host-microbiota interaction studies.

### Data availability.

This whole-genome shotgun project has been deposited at DDBJ/ENA/GenBank under the accession number RJZF00000000. The version described in this paper is the first version (RJZF01000000). The BioProject database number of the sequenced strain is PRJNA379379. This project is available under the Sequence Read Archive number SRP168361.
